# Pre-emptive living donor kidney transplantation: A public health justification to change the default

**DOI:** 10.3389/fpubh.2023.1124453

**Published:** 2023-03-17

**Authors:** Isaac Kim, Umberto Maggiore, Simon R. Knight, Reshma Rana Magar, Liset H. M. Pengel, Frank J. M. F. Dor

**Affiliations:** ^1^Imperial College Renal and Transplant Centre, Hammersmith Hospital, Imperial College Healthcare NHS Trust, London, United Kingdom; ^2^Dipartimento di Medicina e Chirurgia, Università di Parma, Unità Operativa Nefrologia, Azienda Ospedaliera-Universitaria Parma, Parma, Italy; ^3^Sir Peter Morris Centre for Evidence in Transplantation, Nuffield Department of Surgical Sciences, University of Oxford, Oxford, United Kingdom; ^4^Department of Surgery and Cancer, Imperial College London, London, United Kingdom

**Keywords:** living donor, pre-emptive, kidney transplantation, public health, kidney replacement therapy (KRT), evidence based healthcare, dialysis, end-stage kidney disease (ESKD)

## 1. Introduction

Kidney transplantation (KT) is the preferred kidney replacement therapy (KRT) for suitable patients with end-stage kidney disease (ESKD) (1). Donor kidneys could be from a deceased donor (DD) or a living donor (LD). LD kidney transplantation (LDKT) is preferred over DD kidney transplantation (DDKT), because of superior quality kidneys that result in improved patient and graft survival (2), greater flexibility for transplantation across the ABO (3, 4) and HLA (5, 6) barriers, and the possibility for kidney exchange (7) including chains initiated by unspecified donors (8). Perhaps the most important advantage of LDKT is the ability to plan the transplant and hence avoid dialysis, thereby offering the most secure way to achieve pre-emptive KT (PKT). This is not entirely possible with DDKT which may occur too early or too late with respect to the onset of ESKD in an era of continuing shortage of DD organs (9).

The very first successful KT in 1954 (10) which took place 6 years before haemodialysis became available as KRT (11) was in-fact pre-emptive.

PKT has many advantages over non-pre-emptive KT (nPKT) and should be considered for all patients eligible for KT. These include longer patient and graft survival (12–15) and avoiding the risks, complications and restrictions of dialysis. Despite these evidence-based advantages of PKT, both in adult (12, 15) and paediatric (13, 14, 16) patients, the clinical reality is that pre-emptive LDKT (PLDKT) rates are disappointing, even in countries with high rates of LDKT, as it is not used as a quality indicator in most countries. This is worthy of discussion, especially if parallels are drawn to other clinical fields such as oncology, where best treatment options according to the latest evidence are strived for.

In this article, we seek to explore what the justifications may be to promote a greater proportion of our patients undergoing PLDKT, and thus advocate for drastic pathway changes to make PLDKT the default KRT that clinical teams should be delivering on.

## 2. Background

The barriers towards PLDKT have been widely discussed in the literature (17, 18). These include late referral, lack of cohesion, lack of education and insufficient infrastructure and financial support.

Historically, there have been several theoretical drawbacks to PKT raised. These include earlier exposure to the risks of immunosuppression and transplantation surgery (19, 20), potential earlier loss of residual native kidney function and higher risk of non-adherence to immunosuppressants due to not having experienced the morbidity of dialysis (12). The latter fear however has since been disproven (21).

The most common pathway in the United Kingdom (UK) remains DDKT after starting dialysis (22). The UK Renal Registry 24th Annual Report showed that only 17% of all KRT starters are listed or receive LDKT before starting dialysis (23). Between April 2021 and March 2022, only 40% of adult kidney only transplants were from LDs (22) and only 35% of these transplants were pre-emptive (24). In comparison, 50% of kidney transplants in the Netherlands in 2021 were from LDs and a greater proportion of these patients (44%) were pre-emptive (25).

In the UK, median waiting time from start of dialysis to DDKT was 1,044 days for adults transplanted between April 2021 and March 2022 (22). There is substantial mortality on dialysis (26), in addition to a negative impact on employment (27), societal participation (28) and quality of life (QOL) (29, 30). Dialysis also leads to considerable healthcare costs (31). Patients also face a significant risk of suspension from the waiting list (WL) with associated increased mortality and worse graft outcome (32). In comparison to DDKT waiting times, the process of working up an LD to secure PKT is considerably shorter at 90–120 days in the UK (33).

## 3. Guidelines

Currently, there is limited guidance with regards to PLDKT. Most guidelines recommend LDKT over DDKT but do not comment on PKT (34–37).

The position statement by the Descartes Working Group and the European Renal Best Practise Advisory Board provides strong recommendations in support of PKT and PLDKT (38).

“Guidelines for Living Donor Kidney Transplantation” published jointly by the Renal Association and British Transplantation Society from March 2018 states that “kidney transplantation from a living donor, when available, is the treatment of choice for most patients with end-stage kidney disease” and that “the goal should be pre-emptive transplantation” (39). With regards to children with ESKD, it states, “pre-emptive living related renal transplantation is the gold standard therapy.”

The National Institute for Health and Clinical Excellence recommends including “living donor transplantation in the full informed discussion of options for RRT” and offering “pre-emptive living donor transplant ... or pre-emptive listing for deceased donor transplantation to people considered eligible” (40). Pre-emptive listing for DDKT however does not necessarily translate into high rates of PKT, given the nature of the allocation systems worldwide.

## 4. COVID

The SARS-CoV-2 pandemic had a profound impact on KT (41) and especially on LDKT with 1,023 fewer adult kidney only transplants being performed in the UK between April 2020 and March 2021 compared to the previous year (42). Only 17% of these transplants were from LDs, compared to 30% the year before, equating to 573 fewer LD kidney transplants. PKT rate was however maintained at 38% and LDKT was more likely to be pre-emptive than DDKT (43).

A similar picture was seen worldwide with KT from LDs decreasing in most countries (44, 45).

## 5. The public health case

### 5.1. Cost

The cost and sustainability of healthcare has never been as important given the increasing age of the worldwide population (46).

When directly compared, PLDKT was found to be a “cost-saving strategy compared with non-pre-emptive KT strategies” (47). Compared to maintenance dialysis, LDKT was associated with cost-savings of $94,579 over a 20-year period in one study (29) and “represented a saving of €13,102.97 per patient/year” with a payback period of <1 year in another (30). In the latter study, 89% of the transplants were pre-emptive with the authors concluding that PKT should be encouraged from a health budget perspective (30). PKT avoids the cost of dialysis, which has been estimated to be between £20,660 to £31,785 per patient per year (31), and its complications completely.

Decreasing the number of patients starting dialysis by virtue of undergoing PKT will reduce the need for dialysis capacity, allowing resources to be reallocated elsewhere. Preventing the burden of having dialysis three times a week, may enable patients to continue to work and contribute to society.

### 5.2. QOL and recipient outcomes

PLDKT is not just cost saving but also beneficial to the patient's QOL and clinical outcomes.

Compared to non-pre-emptive strategies, the quality-adjusted life year (QALY) “gained of PLDKT was 0.47” (47). Furthermore, LDKT when compared with maintenance dialysis added 3.5 QALYs over 20 years (29) and was associated with enhanced QOL (30).

Superior graft and patient survival are seen when comparing PKT to nPKT (12, 14–16). This was the case for both adult (12, 15) and paediatric (13, 14, 16) patients, and well as DDs and LDs (12). This is not surprising, given dialysis vintage prior to transplantation has been demonstrated to negatively impact graft survival and proposed to be the “strongest independent modifiable risk factor for renal transplant outcomes” (48, 49).

### 5.3. PLDKT vs. pre-emptive DDKT

There has been no direct comparison of PLDKT and pre-emptive DDKT (PDDKT) as identified in a recent systematic review and meta-analysis (50).

There is inherent difficulty in achieving PDDKT as there is no guarantee that pre-emptive listing will lead to PKT. With a DD, it is not possible to know in advance when KT will take place. Furthermore, PDDKT poses ethical dilemmas over the allocation of a scarce resource (51).

## 6. Donor risk

An important caveat that cannot be ignored is the risk to an LD. These include the risks of surgery (52), albeit very low complication risks if well selected (53), and the consequences of living with one kidney.

Kidney donors have an increased relative risk of ESKD; however, the magnitude of the absolute risk increase is small, and LDs still have a risk of ESKD that is much lower than the general population due to the screening and selection of healthy individuals (54). There is also an increased risk of cardiovascular and all-cause mortality however the authors concluded that they would continue to promote LDKT despite these findings (55). Finally, the ERA-EDTA DESCARTES working group concluded that “living kidney donation should be regarded as an acceptable procedure, as the long-term risks for the donor are generally low and, in many instances, offset by the overall benefit for both the donor and recipient” (56).

It is important to factor in the risk mitigation that takes place for LDs. This includes thorough workup, a focus on operative and anaesthetic safety and yearly follow up post-donation, all according to clear guidelines (39). Annual follow-up means that potential issues such as diabetes and hypertension may be detected earlier than if the individual had not donated allowing effective management. In some countries, such as the Netherlands and the UK, prioritisation on the WL is given should LDs develop ESKD (57).

LDKT, being a planned procedure enables a more thorough work up of the donor, reducing the risk of transmission of infection and/or cancer to the recipient (33). For the healthcare organisation, it permits greater control over theatre, bed and workforce availability. For those without an LD option, increasing LDKT rates will increase the availability of DD kidneys.

## 7. Equitable access

The UK National Health Service states that “public health contributes to reducing the causes of ill-health and improving people's health and wellbeing through … ensuring that our health services are most effective, most efficient and equally accessible” (58).

We have so far presented the case for PLDKT as the most effective and efficient form of KRT from a public health point of view. What follows is ensuring PLDKT is equally accessible.

The Getting It Right First Time programme national specialty report for renal medicine recommended reducing “unwarranted variation in deceased and living donor transplantation” (1). Inequity in access to KT (59) clearly exists. There is a 22% increase in time to being wait-listed and a 47% increase in time to LDKT for patients of low education level (60). Wide variation was seen in pre-emptive listing rates across centres (59). There is further variation according to age, ethnicity and socioeconomic status in the UK (61, 62) with older age and body mass index of >35 lowering the likelihood of pre-emptive listing (59).

Suggestions to improve PKT rates can be found in the literature. Early referral (63) is vital in ensuring there is time to sort out the logistics of PKT and LD work up. The timing of this referral should take into consideration the individual recipient's circumstances including rate of renal function decline and disease progression (39) rather than being defined by a specific level of renal function alone. Sufficient time should be allowed for patients to discuss donation within their social network to identify potential LDs.

Education to empower the patient through this process is essential (64, 65). A change in healthcare policy to reduce dialysis capacity and increase transplantation capacity have also been put forward (66).

One example of action being taken is the peer phone buddy scheme by the Gift of Living Donation organisation in the UK which seeks to “provide Black African Caribbean patients with … information about living kidney donation from people from their own community who have lived experience of living kidney donation” (67).

Another is the Kidney Team at Home intervention in the Netherlands which has been shown to be a cost-effective way of significantly increasing LDKT (68, 69). This involved group educational intervention of the patient and the patient's social network, in the patient's home.

Although the focus of these interventions is LDKT, strategies to successfully increase PKT largely rely on maximising LDKT.

## 8. Summary and conclusion

The drastic changes required cannot be understated. The shift from current practice, which sees DDKT after starting dialysis to PLDKT will significantly impact and challenge healthcare systems and practices.

The UK is clear that PLDKT is a major objective as set out in the “Organ donation and transplantation 2030: meeting the need” strategy (70). Another key objective is a more sustainable and reliable system. PLDKT due to its elective nature fulfils these criteria. Although PLDKT cannot fully replace DDKT, maximising its potential would reduce the need to recondition DD organs with expensive technology and infrastructure.

The evidence strongly supports PLDKT as the treatment of choice for ESKD. It is therefore our duty, not only for the individual ESKD patient, but also from a public health perspective, to urgently deliver PLDKT on a much larger scale as the default and in an equitable fashion, and for this to be used as a quality indicator.

## Author contributions

IK and FD conceptualised and wrote the article. UM, SK, RR, and LP reviewed and revised the article. All authors contributed to the article and approved the submitted version.
